# Gut-Lung Microbiota in Chronic Pulmonary Diseases: Evolution, Pathogenesis, and Therapeutics

**DOI:** 10.1155/2021/9278441

**Published:** 2021-12-03

**Authors:** Chang Yi Shi, Chen Huan Yu, Wen Ying Yu, Hua Zhong Ying

**Affiliations:** ^1^Zhejiang Provincial Laboratory of Experimental Animal's & Nonclinical Laboratory Studies, Hangzhou Medical College, Hangzhou, China; ^2^Institute of Cancer and Basic Medicine, Chinese Academy of Sciences, Hangzhou, China; ^3^Cancer Hospital of the University of Chinese Academy of Sciences (Zhejiang Cancer Hospital), Hangzhou, China

## Abstract

The microbiota colonized in the human body has a symbiotic relationship with human body and forms a different microecosystem, which affects human immunity, metabolism, endocrine, and other physiological processes. The imbalance of microbiota is usually linked to the aberrant immune responses and inflammation, which eventually promotes the occurrence and development of respiratory diseases. Patients with chronic respiratory diseases, including asthma, COPD, bronchiectasis, and idiopathic pulmonary fibrosis, often have alteration of the composition and function of intestinal and lung microbiota. Gut microbiota affects respiratory immunity and barrier function through the lung-gut microbiota, resulting in altered prognosis of chronic respiratory diseases. In turn, lung dysbiosis promotes aggravation of lung diseases and causes intestinal dysfunction through persistent activation of lymphoid cells in the body. Recent advances in next-generation sequencing technology have disclosed the pivotal roles of lung-gut microbiota in the pathogenesis of chronic respiratory diseases. This review focuses on the association between the gut-lung dysbiosis and respiratory diseases pathogenesis. In addition, potential therapeutic modalities, such as probiotics and fecal microbiota transplantation, are also evaluated for the prevention of chronic respiratory diseases.

## 1. Introduction

With the development of high-throughput second-generation sequencing technology and through the analysis and sequencing of the whole gene spectrum of microbiota, a certain correlation between the respiratory tract and the intestine has been gradually found [1, 2], and certain microbiota disorders or microbial pathogens in the lungs and intestines have been discovered to be capable of affecting the occurrence, development, and prognosis of diseases through different means, such as inflammation, metabolism, and cell signaling [3, 4]. Clinically, lung diseases, such as asthma, chronic obstructive pulmonary disease (COPD), and even lung cancer, are often associated with digestive tract diseases, resulting in prolonged disease courses, aggravated diseases, and increased mortality [5–8].

In these circumstances, the concept of the lung-gut axis was put forward in modern medicine. This theory uses the immune system and microbial flora, which colonize in the lung and gut, as a link hub to form a two-way axis that connects the lungs and intestines; in other words, intestinal flora influences the development of lung diseases, and in turn, lung diseases, especially infectious diseases caused by various bacteria, can also affect the digestive tract through immunoregulation. The lung-gut link proposed by the lung-gut axis provides a new insight for clinical diagnosis and treatment of the lung diseases through modulating the intestine system and vice versa. This link further explains the scientific nature of the concept of the “exterior-interior relationship between the lung and the large intestine” in Chinese medicine. In this study, the progress of research on the lung-gut axis and the effects of lung and intestinal microecology on lung diseases are reviewed and surveyed.

## 2. Interaction between Lung and Intestinal Microbiota

A large and varied number of microorganisms live in the human body and are mainly distributed on mucosal surfaces, such as the oral cavity, intestinal tract, respiratory tract, skin, and vagina, forming a highly complex microecosystem [9–11]. Moreover, the numerous and various microorganisms in different parts of the body not only help the human body to maintain normal physiological functions but also play an important role in the occurrence and development of disease.

### 2.1. Gut Microbiota and Respiratory Diseases

At present, more than 1000 kinds of intestinal flora are known. They mainly include *Bacteroides*, *Firmicutes*, *Actinomycetes*, and *Verrucomicrobia* [12, 13]. Gut flora consists of approximately 38 trillion bacteria, which can encode approximately 3.3 million specific genes [14, 15]. Each microbiome is distributed in different parts of the gastrointestinal tract in accordance with pH gradient and oxygen content.

Intestinal flora is not only involved in the immune development of the intestinal mucosa but also known as an important innate immune system regulator. Research has found that the development of the immune system is greatly affected by gut microbes [16–18]. In the early stage of life, the incidence of immune system diseases, asthma, and other allergic diseases is significantly increased due to the lack of the irritation of gut microbes [19–22]. This incidence shows the trend of being higher in developed countries than in developing countries and in cities than in rural areas. This trend is related to the reduction in intestinal flora diversity due to the improved hygiene and good medical conditions in developed countries and cities. Epidemiological studies have also confirmed that the use of broad-spectrum antibiotics in infants and young children reduces the variety of gut microbes [19, 23–25]. This effect is a contributing factor to allergic asthma in adulthood [26–28]. Therefore, intestinal flora, especially those in early life, have an important effect on the development of immune system diseases and respiratory diseases.

### 2.2. Lung Microbiota and Respiratory Diseases

**Figure 1:**
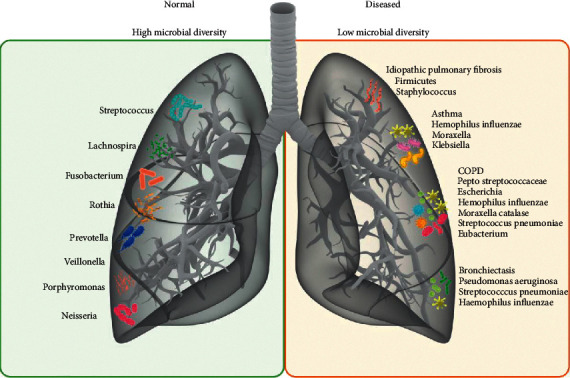
The profiles of lung microbiota in the lung tissues of healthy people and the patients with various chronic pulmonary diseases.

**Figure 2:**
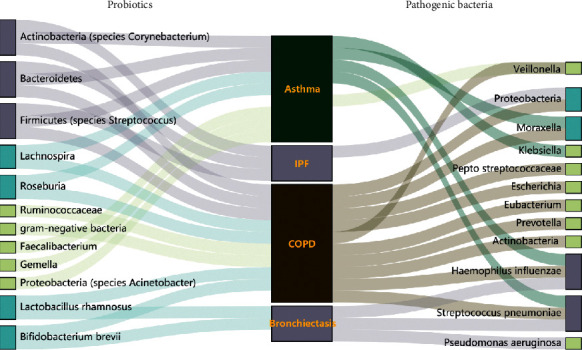
Sankey diagram of gut-lung microbiota composition at genus or species level during the development of various respiratory diseases.

Up to now, less is known about lung microecology than about intestinal microecology. In a healthy state, *Prevotella*, *Streptococcus*, *Veronococcus*, *Fusobacterium*, and *Haemophilus* are the dominant bacteria in the human respiratory tract and lungs [29], but their relative abundances are remarkably less than those in the intestine (Figure 1). It has been proven that the lung-based microorganisms play the biologic roles primarily through regulation of the immune system [10, 30, 31]. In the early stage of life, lung microorganisms migrate into the lungs from pharyngeal secretions or gastric juice mainly through microaspiration and finally are removed through phagocytosis by alveolar macrophages and transported by mucociliary cilia, thereby promoting the maturation of the immune system to achieve the balance and stability of lung microecology. However, in the state of disease, microbial homeostasis in the lungs is disturbed due to the following: (1) changes in the respiratory tract environment caused by chronic inflammation are conducive to the growth and reproduction of certain flora (Figure 2). It is now clear that the number of *Pseudomonas aeruginosa*, *Staphylococcus*, and *Burkholderia* is significantly increased in the respiratory tracts of patients with cystic fibrosis. In patients with COPD, the number of *Moraxella* and *Haemophilus* bacteria in the lungs is increased [32, 33]. The bacteria from the genera *Fusobacterium*, *Lachnospira*, *Veillonella*, and *Rothia* are more common in asthmatic patients than in healthy [29]. The supplementation of these genera in nude mice can reduce the number of pulmonary eosinophils, reduce the immune response of Th1/Th2 or Th17 [34–36], and alleviate the symptoms of those abovementioned respiratory diseases [37–39]. Notably, *Haemophilus influenzae*, *Moraxella catarrhalis*, *Streptococcus pneumoniae*, and *Klebsiella pneumoniae* were found to be the most common bacterial species in patients with severe respiratory diseases, which were also considered to be the potential pathogenic factors [40–42]. (2) The pulmonary epithelial barrier dysfunction impacts the removal mechanism for lung microorganisms (e.g., damaged mucosa cilia) or promotes the migration of microorganisms to the lungs (e.g., secondary infections). Although the mechanism of action through which lung flora influence the development of disease is not clear, it can be used as a potential target for the diagnosis and treatment of diseases and provide a new basis for reasonable disease classification and thus has a good clinical application value.

### 2.3. Mucosal Immunity Bridges the Lung-Gut Axis

From the perspective of embryonic development, the lungs, trachea, and large intestines are homologous, in which the alveolar, glandular, and mucosal epithelia all develop from the endoderm of the archenteron. The mucosal structure of the respiratory tract and gastrointestinal tract is not only an important site for the survival of microflora but also protects the body from pathogen invasion through the mucosal immune system [43, 44]. The physiological conditions on the surface of the mucosa, such as temperature, humidity, and pH, as well as secretions, can affect the growth and migration of microorganisms. In addition, immunoglobulin sIgA, which is secreted by the mucosa, has a selective effect on microorganisms on the surfaces of the mucosa. For example, some pathogens are removed by binding to sIgA, whereas some nonpathogenic and beneficial bacteria can be retained on the mucosal surface by binding to sIgA. Moreover, the body's own congenital immunity and adaptive immunity also play a regulatory role in microecology. The immune system can use inherent immune cells or epithelial cells to identify the presence of microbes and release antimicrobial peptides (such as *α*-defensins) and inflammatory factors to further activate lymphocytes to produce an immune response. In addition to endogenous factors, such as mucosal properties and the immune system, exogenous factors, such as diet structure, glucocorticoids, antibiotics, lifestyle, and environment, can affect the composition and function of bacteria in the lung and gut microbiota [45–47].

## 3. The Lung-Gut Microbiome Crosstalk

**Figure 3:**
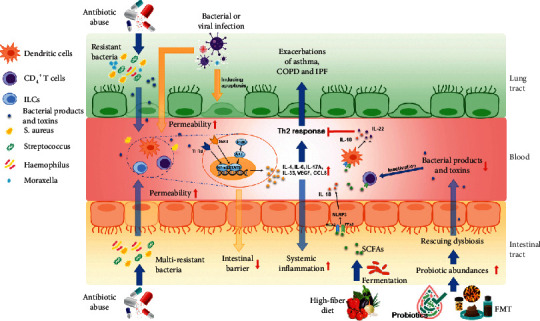
The role of lung and gut microbiota in the pathology of respiratory diseases.

The intestines and lungs interact with and restrict each other through microorganisms, immune functions, and metabolites, thus achieving two-way regulation (Figure 3).

### 3.1. Direct Interaction between Lung and Gut Microbiome

The microorganisms that have colonized the mucosa of the respiratory and digestive tracts can have a regulatory effect on tissues and are the material basis for lung-gut connections. For example, gavage with a suspension of feces from healthy mice can alleviate the symptoms of pneumonia in mice infected with *Streptococcus pneumoniae* under antibiotic treatment [48, 49]. In children, oral administration of *Lactobacillus* and *Bifidobacterium* can help relieve asthma symptoms and reduce the frequency of seizures [50]. These results have shown that changes in gut microbes can cause changes in lung immunity and lung diseases. Conversely, *S. pneumoniae* and *Haemophilus* flu in the lungs activate the MAPK pathways of intestinal tissue cells and enhance the inflammatory response [51–53]. In addition, gut microbes can be transferred to the lungs [54]. For example, the deterioration of sepsis and acute respiratory distress syndrome has been clinically found to be promoted when the integrity of the intestinal mucosa is destroyed, causing the intestinal flora to transfer into blood and even the lungs [55, 56].

### 3.2. Immunomodulation of Lung and Gut Microbiome

Studies have shown that certain lung and intestinal flora can affect the body's immune system. For example, segmented filamentous bacteria in the gut can stimulate the body to produce Th17 immune cells, thus reducing the infection rate and mortality rate of *S. pneumoniae* [57, 58]. In mice, gut inoculation with *Lactobacillus johnsonii* can significantly reduce the inflammatory response of Th2 in the lungs [59]. In addition, when intestinal or lung flora disorders occur in the body, immune cells, such as ILC2s, can migrate through blood in the lungs and intestines, releasing excessive inflammatory media and thus affecting the microecological environment of the lungs and the type and intensity of the immune response.

### 3.3. Gut Microbiota Metabolites and Respiratory Diseases

Certain components or metabolites of gut microbiota, such as short chain fatty acids (SCFAs) [60, 61], lipopolysaccharide (LPS), and peptide peptidoglycans, also play an important role in the body when it is in a diseased or healthy state [17, 62, 63]. Studies on SCFA functions are the most detailed. SCFAs in the intestinal lumen provide energy to colon cells and regulate immune response in the intestine to maintain the stability of the intestinal microecology. In addition, SCFAs can activate downstream effect molecules (e.g., MAPK, PI3K, and NLRP3) by binding to G protein-coupled receptors (e.g., GRP43, FFA2, and HCA2) on cell membranes, thus changing dendritic cells (DCs) and auxiliary T cells [64], which can also enter the cell via the transporters SLC5A8 or SLC16A1 [65, 66], inhibit the activity of histone deacetylase, and increase the number of Ly6c− monocytes in the bone marrow and lungs, thereby reducing the production of neutrophils and improving allergic inflammation in the lungs. In addition to SCFAs, metabolites produced by intestinal flora, such as desaminotyrosine, indole derivatives, niacin, polyamine, urolithin A, pyruvate, and lactic acid, have anti-inflammatory and antiinfection activities. For example, indoles and indoles' derivatives can inhibit central nervous system inflammation by activating aryl hydrocarbon receptor signaling in astrocytes and regulate intestinal ecosystem function, thus playing anti-inflammatory and antioxidant roles [67, 68].

## 4. Probiotics and Fecal Microbiota Transplantation for Treatment of Respiratory Diseases

Given that intestinal flora play an important role in the human body, attempts have been made to treat diseases with complementary probiotics (mainly composed of *Bifidobacteria* and *Lactobacilli*) [23–25] or fecal microbiota transplantation [48, 49].

It was reported previously that 6 h after FMT, the pulmonary bacterial counts as well as TNF-*α* and IL-10 levels were remarkably normalized in microbiota-depleted mice, indicating the protection of gut microbiota against pneumococcal pneumonia [18]. Similarly, FMT downregulated the activity of the TLR4/NF-kB signaling pathway and relieved oxidative stress in animals with acute lung injury by restoring the gut microecology [69]. They can not only treat all kinds of intestinal diseases caused by intestinal flora disorders but also have a positive effect on the prevention and treatment of infectious diseases. In particular, the clinical treatment guidelines made by the United States, China, and other countries for the prevention of COVID-19 pneumonia have clearly proposed that intestinal microecological regulators can be used to maintain intestinal microbiota hemostasis and prevent secondary lung infection [70, 71]. However, a certain risk for pathogenic bacterial contamination, which can increase the occurrence of immune-related adverse events, may exist regardless of the use of flora regulation agents or flora transplantation. Therefore, in clinical practice, we should pay attention to the safety and quality control of microflora regulation agents or flora transplantation and prevent and reduce the occurrence of adverse events as much as possible while enhancing efficacy.

## 5. The Immunomodulation of Traditional Chinese Medicine on Lung Dysbiosis

The theory of the exterior-interior relationship between the lung and large intestine is an important part of the Tibetan elephant theory in traditional Chinese medicine. As early as 3000 years ago, the classic Huangdi Neijing of traditional Chinese medicine recorded the physiological and pathological relationship between the lungs and the large intestine in detail. Xuanbai Chengqi decoction, Gegen Qinlian decoction, and other tonic Chinese medicines, such as Ginseng Radix et Rhizoma, Gardeniae Fructus Praeparatus, Angelicae Sinensis Radix, and Astragali Radix, can improve LPS-induced acute lung tissue damage and pathological colon tissue damage by adjusting the lung-gut mucosal immune function and are thus candidate drugs in innovative drug development based on the concept of treating the lung and intestine together [72–74].

However, the current research on the mechanism of traditional Chinese medicine has mainly focused on the changes in the expression levels of secreted IgA and cytokines and the number of immune cells, such as T lymphocytes. In-depth studies on airway/intestinal mucus secretion, changes in immune cell function in mucosal systems, and changes in the local microecological components of the lung-gut axis are lacking.

## 6. Future Challenges and Prospects

With the further development of microbiome research, people have increasingly realized the important role of lung and gut microecology in the body, and the mechanism behind the lung-gut axis has been gradually uncovered in many clinical phenomena and experimental data. However, due to the differences in the sources of clinical trial samples, the consistency and repeatability of the results are poor. Given the lack of longitudinal or intrusive research on the microbiome, the study of the specific mechanisms and pathways of the gut-lung axis remains difficult, and oral probiotic administration, flora transplantation, or antibiotic prevention and treatment still need further verification. In the future, with the updating of sample-handling methods, advances in biotechnology, and increased interpretation of sequencing results, this area could lead to revolutionary advances in the prevention and treatment of lung diseases and provide new ideas and therapeutic targets for the clinical treatment of related diseases.

## Data Availability

No data were used to support this study.
